# Editorial: Self in the space-time continuum: from basic perception to complex social cognition

**DOI:** 10.3389/fpsyg.2023.1198227

**Published:** 2023-05-09

**Authors:** Jianqiao Ge, Weiwei Sun, Bin Zhou

**Affiliations:** ^1^Academy for Advanced Interdisciplinary Studies, Peking University, Beijing, China; ^2^Department of Computer Science and Technology, University of Cambridge, Cambridge, United Kingdom; ^3^Institute of Psychology, Chinese Academy of Sciences, Beijing, China; ^4^Department of Psychology, University of Chinese Academy of Sciences, Beijing, China

**Keywords:** self, self-awareness, embodiment, self-representation, self-reference, episodic memory

The sense of self is generally considered as the core of our mental life, yet its underlying mechanism remains unclear. From both phenomenological and functional perspectives, self is experienced as a spatially constrained entity and temporally extended continuum, and self-related operations can unfold across a wide range of hierarchically structured processing stages from basic perception to complex social cognition. It has been argued that the interoceptive and visceromotor signals, which underlying the communication between the internal organs and the brain, provide biological basis of the embodied self as well as the self-entity in space (Seth, 2013). Nevertheless, the construct of self involves broader multisensory and motoric integration and mapping, as indicated by the phenomenon of mirror self-recognition (Anderson and Gallup, 2015) and illusions such as rubber hand illusion (Botvinick and Cohen, 1998) and out-of-body experience (Blanke and Arzy, 2005). How the neural system coordinates signals arising from both internal (e.g., cardiac) and external (e.g., visual) domains to generate a feeling of self remains a big challenge for the future research. Furthermore, our sense of self is apparently not stuck in the present, but can rather flexibly travel to the deep past and far future through abilities such as mental time travel. We often observe ourselves in episodic memories as our own doppelgangers, a feeling profoundly unlike our present selves experienced from a first-person perspective. All these complexities encourage us to develop unified concepts to encompass various spatiotemporal aspects of self from perspectives such as homeostasis-dependent entity construction (Zhou et al., 2014) and torso-centered signal integration (Park and Blanke, 2019) as well as some contributions in the current Research Topic (Garcia-Pelegrin et al.).

Modern advances in physics have proposed to unify the dimensions of space and time into a single entity, known as the space-time continuum, to regard a particular object as a representation of its space-time union. A potentially promising direction for scientific research is to similarly regard the self as a combined entity of both its spatial and temporal constructs. With such perspective in the horizon, important insights have been gained from research on topics such as the embodied self and self-image in space, and self-reference and time travel in the autobiographic memory. There are also thoughts and studies probing the cognitive, computational, and neural mechanisms underlying the sense of self based on different scales of spatial and temporal processes. Another important progress has been also made concerning the evolutionary bases of the sense of self, and the uniqueness of human mind. Nevertheless, it remains to be elucidated what is the prerequisite cognitive machinery that supports the occurrence of self-experiences and which animals or artificial intelligence possess such abilities.

Contributions in this Research Topic touched such relevant questions in both empirical research and theoretical considerations. Their work provided important evidence and insights for us to examine various processing stages and different spatial and temporal aspects in the experience of self, thus advancing toward a more comprehensive and deeper understanding of the mechanism of self.

One article showed that the degree of the full body illusion (FBI), in which the sense of body ownership is shifted to an object (e.g., avatar) located in another place, is modulated by the top-down cognition of self-association and also related with the tendency of individual depersonalization (Yamamoto and Nakao). Another article investigated the influence of interoception played in the processing of self-associated external stimuli (Honda and Nakao). These articles together suggest the multisensory nature of constructing an embodied self, highlighting the important involvement of both exteroceptive and interoceptive perception as well as high-level cognitive modulation and individual trait in the processing of sense of self. In addition to these more spatially oriented studies, another article discussed the role played by the art of storytelling in the transcendence of self and experience across the time (*via* mental time travel) space (*via* theory of mind) continuum as well as the evolutionary basis, thus provided an important conceptual contribution to our understanding of self and its potential timeline in the evolution (Garcia-Pelegrin et al.). Interestingly, by showing self-reference effect with better recall performance on items that were presented instantaneously after their own-names for high performers, one article expanded our exploration on self-representation in unconsciousness (Yaoi et al.). Further, in regard of the sense of wholeness and continuity of the self, a contributing article proposed theory for the neurobiological basis of such sense, and hypothesized that the self-continuity was maintained by two principles: the principle of reafferent (or corollary discharge) and the principle of a time theory (Izadifar). Intriguingly, this article also raised the possible existence of a precise temporal integration mechanism in the internal neural system, which works with the outside space-time world as the foundation for our uninterrupted experience of the self.

The contributing articles in the Research Topic added new insights in the research about the spatiotemporal processes associated with the self-representation, thus provided a picture of, though not yet complete, the evolutionary and functional basis of the occurrence of sense of self.

## Author contributions

JG, WS, and BZ conceptualized the study and wrote the paper. All authors contributed to the article and approved the submitted version.

## References

[B1] Anderson J. R. Gallup G. G. (2015). Mirror self-recognition: a review and critique of attempts to promote and engineer self-recognition in primates. Primates 56, 317–326. 10.1007/s10329-015-0488-9 26341947

[B2] Blanke O. Arzy S. (2005). The out-of-body experience: disturbed self-processing at the temporo-parietal junction. Neuroscientist 11, 16–24. 10.1177/1073858404270885 15632275

[B3] Botvinick M. Cohen J. (1998). Rubber hands ‘feel' touch that eyes see. Nature 391, 756. 10.1038/35784 9486643

[B4] Park H.-D. Blanke O. (2019). Coupling inner and outer body for self-consciousness. Trends Cogn. Sci. 23, 377–388. 10.1016/j.tics.2019.02.002 30826212

[B5] Seth A. K. (2013). Interoceptive inference, emotion, and the embodied self. Trends Cogn. Sci. 17, 565–573. 10.1016/j.tics.2013.09.007 24126130

[B6] Zhou B. Pöppel E. Bao Y. (2014). In the jungle of time: the concept of identity as a way out. Front. Psychol. 5, 844. 10.3389/fpsyg.2014.00844 25120528 PMC4114202

